# The Current Landscape of Ophthalmology Training in Europe

**DOI:** 10.7759/cureus.103487

**Published:** 2026-02-12

**Authors:** Maria Sidiropoulou

**Affiliations:** 1 Ophthalmology, UNC Health, Chapel Hill, USA

**Keywords:** entrustable professional activities, european training requirements, ophthalmology residency, ophthalmology training, surgical skills-based training

## Abstract

Ophthalmology training across Europe has undergone a significant transformation over the last two decades. Although individual countries retain distinct systems for resident recruitment, training structure, and surgical exposure, the overarching goal has been to converge toward competency-based education guided by the European Board of Ophthalmology (EBO) and the European Union of Medical Specialists (UEMS).

This narrative review draws on official EBO and UEMS policy documents, recent peer-reviewed literature, national curricula, and surveys of residents and educators. It examines the structure and duration of training, curriculum reforms, surgical exposure, simulation, fellowship opportunities, and the influence of European legislation on professional mobility.

Residency programs in Europe vary in length from four to six years, with considerable heterogeneity in surgical case volume, fellowship opportunities, and access to simulation. The 2024 European Training Requirements (ETR) introduced Entrustable Professional Activities (EPAs), programmatic assessment, and e-portfolios, providing a common reference for national curricula. The EBO Diploma (EBOD) serves as a recognized benchmark for harmonization. Simulation-based cataract training has moved from optional to integral, though adoption remains inconsistent. Pressures such as the European Working Time Directive (EWTD) and the COVID-19 pandemic have altered clinical exposure, while digital education and mobility programs offer compensatory mechanisms.

European ophthalmology training is moving toward harmonized outcomes but retains structural variation between countries. Achieving consistency will require aligning national curricula with the ETR, mandating simulation milestones, investing in faculty development, and formalizing fellowship standards to ensure equitable access and readiness for independent practice.

## Introduction and background

Training in ophthalmology within Europe occupies a unique position in medical education. On the one hand, individual countries retain sovereignty over the recruitment, curriculum, and licensing of ophthalmologists. On the other hand, ophthalmology has been at the forefront of continental harmonization, with the European Board of Ophthalmology (EBO) and the European Union of Medical Specialists (UEMS) Ophthalmology Section promoting shared standards since the early 1990s [1].

The need for harmonization stems from multiple pressures. Free movement of labor within the European Union, the globalization of healthcare recruitment, and rising patient expectations for standardized quality of care all require that an ophthalmologist trained in one country is demonstrably competent to practice safely in another. Moreover, ophthalmology is highly technology-driven, with rapid innovation in surgical devices, imaging, and therapeutics necessitating frequent curriculum renewal.

While progress has been made, particularly with the introduction of the European Training Requirements (ETR) and the growth of the EBO Diploma (EBOD) examination, challenges remain [2,3]. National programs vary widely in length, entry route, and surgical exposure, creating inequities in readiness for independent practice. Simulation technology, increasingly recognized as a cornerstone of surgical education, is unevenly distributed. Fellowships, now a de facto requirement for subspecialization, lack standardization in structure and transparency.

This review provides a comprehensive overview of the current landscape of ophthalmology training in Europe. It focuses on the regulatory framework, curriculum reforms, surgical training, system-level pressures (such as the European Working Time Directive (EWTD) and COVID-19), fellowship opportunities, mobility, and future directions.

## Review

Regulatory architecture and harmonization tools across the EU

The EBO

The EBO was established in 1992 as the permanent working group of the UEMS Ophthalmology Section, with a mission to oversee the standards of education in ophthalmology across Europe and to harmonize training outcomes [1]. The EBO pursues this mission through four main functions: (1) accreditation of training centers, ensuring quality and comparability across institutions; (2) oversight of continuing medical education (CME), promoting lifelong learning; (3) provision of grants and exchange programs, supporting mobility and equity in training opportunities; and the the EBOD examination [3], a knowledge- and skills-based assessment intended as a supranational benchmark.

The EBOD Examination

The EBOD examination, first introduced in 1995, is now taken by over 700 candidates annually. Although passing EBOD does not confer a license to practice (licensure remains the prerogative of national authorities), it is increasingly recognized by employers and national societies as a symbol of standardized competence [1,4,5] and has grown from a voluntary credential to a widely sought-after qualification. It consists of a written multiple-choice paper and an oral viva structured around clinical scenarios [3]. Blueprinting ensures that the exam covers all domains of the ETR, and psychometric analysis supports defensibility. Many countries now recommend or incentivize EBOD participation; some employers consider EBOD success in selection for fellowships or consultant posts. Its portability and pan-European recognition make it particularly valuable for early-career ophthalmologists seeking cross-border opportunities. Although optional, the EBOD has become an increasingly common component of career progression and a symbol of a move towards harmonized standards between EU countries.

The ETR

The most significant regulatory development has been the publication of the 2024 ETR in Ophthalmology [2]. Developed jointly by UEMS and EBO, the ETR provides a pan-European framework for residency training. The ETR does not seek to homogenize national curricula but provides a shared outcomes framework, enabling cross-border comparability and mobility. It is structured around the following: (1) Entrustable Professional Activities (EPAs), specific clinical and surgical tasks that residents must be able to perform independently by graduation, such as uncomplicated cataract extraction, management of acute glaucoma, or recognition and triage of retinal detachment; (2) competency domains, covering medical knowledge, clinical decision-making, communication, professionalism, and systems-based practice; (3) assessment tools, including structured workplace-based assessments (WBAs), simulation outcomes, and multi-source feedback; and (4) training infrastructure, emphasizing e-portfolios, logbooks, and faculty calibration in assessment.

Professional Mobility and EU Legislation

Harmonization of training is reinforced by the European law. The Directive 2005/36/EC on the recognition of professional qualifications establishes automatic recognition of medical specialties within the EU, facilitating professional mobility of ophthalmologists between member countries [6]. While the directive ensures legal recognition of qualifications, it does not guarantee comparable training experiences, underscoring the need for frameworks such as the ETR and EBOD. Language requirements, differences in fellowship culture, and local employment practices still pose barriers, but the directive provides the legal scaffolding for mobility. In practice, EBOD certification often strengthens applications when moving between systems, particularly when surgical numbers or subspecialty exposure differ.

Ophthalmology Entry Pathways, Structure, and Duration of Training Across the EU

Across Europe, ophthalmology training typically begins after completion of a general medical internship or foundation year. Duration ranges from 4 years in Spain to 7 years in the UK, with most countries requiring 5 or 6 years [4,7] (Table 1). Shorter programs often rely heavily on post-residency fellowships for subspecialty exposure, while longer programs integrate subspecialty rotations into residency. Entry routes to ophthalmology training vary, including competitive national examinations (e.g., France, Spain) that allocate residency posts on a country-wide ranking system; hospital-based recruitment with national oversight (e.g., Germany, Italy) that blends institutional interviews with centralized regulation; national ranking with portfolio review and interviews (e.g., United Kingdom); and national ophthalmology residency waitlist enrolling in individual training hospitals (e.g., Greece) with centralized regulation.

**Table 1:** Illustrative comparison of ophthalmology training across selected European countries (as of 2024-2025). Data were compiled from national curricula, EBO surveys, and published literature (approximate values). EBO: European Board of Ophthalmology, EBOD: European Board of Ophthalmology Diploma, OST: ophthalmic specialty training.

Country	Duration	Entry route	Cataract exposure (approx.)	EBOD uptake	Simulation access
UK	7 years (OST)	National portfolio + interview	~350 cases	High	Universal (EyeSi mandatory)
Germany	5 years	Hospital contract	200-300 cases	Moderate	Variable across centers
France	5 years	National ECN exam	250 cases	Moderate	Growing adoption
Spain	4 years	MIR exam	150-200 cases	Increasing	Expanding, not universal
Sweden	5 years	National recruitment	Variable (200+)	High	Regional simulation hubs

Regarding variability between countries, a 2022 comparative analysis showed striking differences in phacoemulsification case requirements: some countries mandate no explicit minimum, while others require up to 350 completed cases [8]. Similarly, access to subspecialties, such as vitreoretinal surgery, pediatric ophthalmology, or ocular oncology, depends on the size and specialization of training hospitals. In smaller countries, trainees may need to rotate regionally or internationally for certain subspecialties.

Curriculum and Assessment

The 2024 ETRs translate broad competencies into EPAs [2]. EPAs operationalize training outcomes by asking: Can this resident be trusted to perform this task independently? EPAs anchor curricula in observable, workplace-relevant activities rather than abstract descriptors. They also facilitate entrustment decisions and progression reviews, including independently performing uncomplicated phacoemulsification, managing acute angle-closure glaucoma, performing primary repair of corneal lacerations, and conducting emergency lateral canthotomy.

National curricula vary in sophistication, but the ETR provides a common model, a mutual framework for countries to adopt. In our view, this shows that Europe as a whole seems to be increasingly moving toward programmatic assessment. This shift likely reflects dissatisfaction with previous models that relied mostly on case numbers or end-of-training exams.

Although the United Kingdom is now outside the EU, its Ophthalmic Specialty Training (OST) Curriculum 2024 has positively influenced EU training [7]. The UK curriculum spans seven years with clearly defined learning outcomes, WBAs, and a Capabilities in Practice (CiP) framework that parallels the EU's EPAs [9,10]. The Annual Review of Competence Progression (ARCP) ensures longitudinal tracking. Simulation training is embedded, and trainees must demonstrate specific milestones before live surgery [11]. The UK example demonstrates that competency-based curricula can be operationalized at scale, offering lessons for continental adoption of the ETR [10]. The UK’s OST 2024 handbook notably describes how CiPs are successfully performed via WBAs and includes simulation outcomes and supervisor entrustment scales [7,12]. Similar structures are being piloted in the Netherlands, Germany, and Scandinavia [13,14].

Systemic Pressures on Training: The Impact of the EWTD

The EWTD, limiting working hours to 48 per week, has reshaped residency training across specialties, including ophthalmology. While it was intended to improve patient safety and physician well-being, it has nonetheless reduced the sheer number of hours trainees can spend in clinics and operating rooms in their given residency timeline [15,16]. Several surveys reveal mixed perceptions. Many residents report fewer surgical opportunities, reduced continuity of patient care, and less time with supervisors [15-18]. Others note that protected schedules encourage efficiency and prevent fatigue-related errors. Importantly, implementation varies: in some countries, EWTD compliance is strict, while in others, flexibility is built into on-call structures. Overall, while the EWTD protects well-being, it has inadvertently reinforced the need for competency-based assessment rather than simple time- or volume-based models. To mitigate reduced exposure time, some programs have developed resident-protected surgical lists to ensure equitable access to cataract and subspecialty cases; short-block intensive modules (e.g., two-week cataract bootcamps) to concentrate exposure; and extended use of virtual reality surgical simulation (EyeSi), particularly for those with limited OR time [19-22].

COVID-19: A Generation-Defying Disruption

The global impact of COVID-19 on ophthalmology resident training has been profound and multifactorial. Clinical and surgical trainings were significantly disrupted worldwide, with many programs reporting reductions of over 50% in clinical activity and over 75% in surgical volume during peak pandemic periods. This led to decreased hands-on experience, particularly in cataract and other microsurgical procedures, which are critical for competency development [23,24]. The pandemic had also caused widespread redeployment of ophthalmology residents to non-specialty COVID-19 duties, further limiting specialty-specific training and contributing to a perceived knowledge and skills gap. In response, many programs rapidly adopted virtual learning modalities, including web-based didactics, surgical video discussions, and simulation-based training, which were positively received and are now considered essential adjuncts to traditional training [23,25-27]. Virtual platforms (such as Zoom and others) became widely used for theoretical teaching, while surgical simulators partially mitigated the loss of operative exposure [23,26,28].

COVID-19-related studies from across Europe documented (1) declines in surgical case numbers (in some centers, residents performed fewer than half their expected cataract cases during 2020-2021); (2) interrupted continuity of subspecialty rotations, delaying acquisition of critical skills; (3) loss of face-to-face teaching and exams, with rapid adoption of online webinars and virtual grand rounds; and (4) psychological stress and well-being challenges, with residents reporting anxiety about delayed competence and career progression [29].

Adaptations included (1) acceleration of digital platforms utilization (webinars, e-learning modules, and case-based teleconferences); (2) expansion of simulation use (EyeSi and wet-lab practice helped maintain skills while elective lists were on hold); and (3) modified assessment (some national exams adopted remote proctoring or adjusted requirements).

In summary, COVID-19 caused a global decline in ophthalmology resident clinical and surgical training, negatively impacted trainee mental health, and accelerated the integration of virtual and simulation-based education. COVID-19 has normalized hybrid education, with remote learning likely to remain integral to future training paradigms [23-25,28,30]. Most societies now run regular online teaching accessible across borders, in a way democratizing access to high-quality education. However, the lost surgical volume potentially created a "hands-on" training gap for the “COVID generation,” with some requiring fellowship extensions to make up for it.

Fellowships and the Transition to Independent Practice in the EU

Across Europe, fellowships have become increasingly crucial for subspecialization and for consolidating surgical skills (especially when residency exposure is limited on a subject). Fellowships range in duration, from one-year cataract posts to two-year vitreoretinal, corneal, and other subspecialty fellowships.

Some of the drivers for fellowship pursuit include (1) variability in exposure during residency, particularly in subspecialties like oculoplastics and pediatric ophthalmology; (2) employer expectations, as many hospitals now prefer or require fellowship training for consultant posts; and (3) individual preference, with many residents seeking international fellowships to gain broader experience.

Fellowship opportunities in ophthalmology are available in most EU countries, primarily within university hospitals and large teaching centers. These fellowships typically focus on subspecialties such as vitreoretinal surgery, glaucoma, cornea and anterior segment, oculoplastics, and pediatric ophthalmology/strabismus. The EBO and national societies support structured fellowships, and subspecialty diplomas are increasingly recognized for advanced training and credentialing [31,32]. International programs, such as those offered by the International Council of Ophthalmology, also provide fellowships, especially for candidates from low-resource settings [33]. However, unlike the ETR for residency, fellowships remain heterogeneous. Duration, surgical volume, assessment, and supervision differ widely. Some fellowships are funded, while others require self-financing, creating inequities. Language proficiency is an additional barrier to accessing cross-border fellowships [31]. The EBO and European Society of Ophthalmology (SOE) surveys have repeatedly highlighted these inequities, noting the need for standardization and transparency [4]. Without reform, fellowships risk perpetuating disparities: well-resourced trainees access competitive international centers, while others remain limited to local options of variable quality.

Calls are also growing for formal fellowship accreditation under a European umbrella, mirroring the ETR for residency. The Fellow of the EBO Subspecialty (FEBOS) exams already exist for cataract, refractive surgery, and retina, offering supranational benchmarks [1,3,32]. Expanding these to other subspecialties and tying them to accredited fellowships would provide standardization and improve transparency for employers and trainees alike.

EU Law and Professional Recognition

The Directive 2005/36/EC guarantees recognition of medical specialist qualifications across the EU [6]. In practice, this facilitates mobility for ophthalmologists once they complete training and licensure. However, mobility during residency or fellowship remains dependent on local regulations, language requirements, and hospital policies. Despite the legal framework, real-world barriers persist: (1) language proficiency, especially in patient-facing specialties like ophthalmology; (2) funding constraints, as not all fellowships or exchanges are salaried; and (3) administrative delays in recognition of training rotations.

In the author's view, these barriers might disproportionately affect residents from smaller or lower-resource countries, who may need mobility the most to access subspecialty exposure.

EBO and SOE Exchange Programs

To reduce inequities, the EBO and SOE run educational exchange programs offering funded observerships and short training visits [1]. These provide valuable exposure but remain limited in scale, relative to demand. Expanding such initiatives could significantly improve equity of access. There are, however, some considerations related to such physician mobility. “Exporting” residents from lower-resource systems to wealthier ones may exacerbate inequities if these physicians choose to not repatriate after training. Conversely, restricting mobility may deny individuals vital opportunities. Balancing these tensions requires carefully designed exchange programs with bilateral commitments.

Research and Scholarship

The modern ophthalmologist must not only provide clinical care but also interpret evidence, contribute to research, and lead quality improvement (QI). The 2024 ETR explicitly includes research literacy and scholarly activity as core competencies [2]. Expectations vary: some countries require completion of a thesis (e.g., Germany), while others emphasize participation in audits and QI projects (e.g., UK). It is a well-established practice for residents to present at national or European meetings, often supported by travel grants. The COVID-19 pandemic positively highlighted the adaptability of research training: residents engaged in big-data studies, registry analysis, and AI projects during reduced clinical activity. We should acknowledge that time constraints might pose a barrier to research, particularly in countries with shorter residencies. Finally, worth noting is the development of teaching skills. Programs such as the International Council of Ophthalmology (ICO) “Teaching the Teachers” initiative equip faculty to deliver competency-based training and reliable assessment [34]. Expanding these across Europe will be critical to introducing and sustaining reforms.

Challenges in European ophthalmology training

Despite advances in harmonization, several persistent challenges shape the landscape of ophthalmology education in Europe.

Heterogeneity in Surgical Exposure

Cataract numbers and access to subspecialties remain highly variable, undermining consistent readiness for independent practice.

Simulation Adoption Gaps

Evidence supports VR and wet-lab simulation uptake is uneven, especially in smaller or resource-limited programs.

Service Versus Education

EWTD compliance and service pressures often reduce protected teaching time and continuity with supervisors.

Fellowship Inequities

Lack of standardization, transparency, and funding creates disparities in subspecialty opportunities.

Mobility Barriers

Language, administrative, and financial obstacles continue to limit cross-border training, despite EU specialty training recognition laws.

Faculty Development

Competency-based education requires trained assessors, yet educator capacity remains underdeveloped.

These challenges underscore the need for continued collective effort at both national and European levels.

Future directions for ophthalmology training

The 2024 ETR defines a clear set of EPAs for residency graduates. Next steps could include (1) national curricula to adopt these EPAs. Optimally, faculty members would need to receive training on making such defensible entrustment decisions. (2) E-portfolios and logbooks should track EPA attainment systematically. (3) Simulation should move from optional to mandatory: gradual introduction of minimum simulator milestones (e.g., EyeSi modules) before live cataract surgery and using simulation data as part of programmatic assessment. (4) While the author acknowledges it as part of a plan that transcends ophthalmology economically and administratively, funding regional training hubs to ensure equitable access would be a huge leap forward for trainees across Europe.

Standardizing fellowships

To address lack of uniformity, fellowships should be (1) accredited by EBO/UEMS with minimum standards for supervision, case exposure, and assessment [1,35]; (2) linked to FEBOS certification, gradually expanding beyond cataract/refractive and retina to other subspecialties [3,32]; and (3) listed in a transparent, ideally multilingual online portal with eligibility criteria, funding details, and outcomes data.

Enhancing professional mobility and equity: training the trainers

Expansion of EBO and SOE exchange grants, possibly prioritizing under-resourced trainees, will help develop bilateral partnerships between training centers and facilitate structured exchanges. EU countries should recognize cross-border rotations within national training requirements. Investing in faculty development and education is also paramount. Competency-based training requires assessors who can deliver reliable feedback and entrustment decisions. Faculty training in WBAs, feedback giving skills, simulator debriefing, and cross-border faculty development initiatives (among others) would only add recognition of their important educational role as the cornerstone of good training.

## Conclusions

European ophthalmology training is steadily transitioning from time-, volume- and service-based models to competency-based frameworks grounded in observable outcomes. The 2024 ETR provides a continental blueprint, while the EBOD examination offers a harmonized benchmark of knowledge and skills. Simulation is becoming central to surgical training, and mobility programs foster shared standards across borders. Yet variability persists in surgical exposure, fellowship opportunities, and educator capacity. Undeniable challenges and limitations present difficulties; different regional laws, healthcare funding disparities between EU countries, facility and equipment availability, and other important variables must be taken into account for future planning of ophthalmology training in the EU as a whole. The author's view is on the importance of steps toward equity, ensuring that every resident, regardless of EU country, hospital size, or resources, can reach a threshold of competence. By aligning curricula with EPAs, mandating simulation milestones, accrediting fellowships, and supporting educators, Europe can deliver a consistent model of ophthalmology training, one that balances national diversity with continental scientific cohesion and prepares specialists for a rapidly evolving clinical and technological future.
